# Stroke Risk After TAVR: Balloon-Expandable vs Self-Expanding Valves in Community Practice

**DOI:** 10.1016/j.jacadv.2026.102727

**Published:** 2026-04-09

**Authors:** Sant Kumar, David Elison, Ashish Pershad, Nezar Falluji

**Affiliations:** a Creighton University School of Medicine, Phoenix, Arizona, USA; b St. Joseph Hospital and Medical Center, Phoenix, Arizona, USA; c Division of Cardiology, Department of Medicine, University of Washington, Seattle, Washington, USA; d Chandler Regional Medical Center, Chandler, Arizona, USA

**Keywords:** balloon-expandable valve, self-expanding valve, stroke, TAVR

## Abstract

**Background:**

Stroke is a serious complication after transcatheter aortic valve replacement (TAVR), yet contemporary data from community hospital practice are limited.

**Objectives:**

The purpose of this study was to evaluate the association between valve type and the risk of stroke within 1 year after contemporary TAVR in community practice.

**Methods:**

We analyzed patients who underwent TAVR across CommonSpirit Health hospitals from January 2021 to February 2023 using data from the Society of Thoracic Surgeons (STS)/American College of Cardiology Transcatheter Valve Therapy Registry. Valve type was categorized as balloon-expandable valves or self-expanding valves (SEV). The primary outcome was stroke within 1 year. Kaplan-Meier methods were used to compare stroke-free survival between valve types. Baseline differences were adjusted using inverse probability of treatment weighting. Independent predictors of stroke were identified using weighted time-to-event models.

**Results:**

A total of 6,663 patients underwent TAVR during the study period; 5,445 (81.7%) received balloon-expandable valve, and 1,218 (18.3%) received SEV. More females received a SEV (56.7% vs 37.5%; *P* < 0.001). The STS risk score (4.5 ± 3.8 vs 4.0 ± 3.5; *P* < 0.001) was higher in the SEV group. A total of 87 (1.3%) patients experienced stroke within the study period. The primary endpoint of stroke-free survival at 1 year was not different between valve types (log-rank *P* = 0.448). After inverse probability of treatment weighting adjustment, valve type was not associated with stroke (adjusted HR: 1.54; 95% CI: 0.79-2.68; *P* = 0.294). Age, lower body mass index, prior stroke, STS risk, and alternative access were associated with stroke.

**Conclusions:**

In this registry of patients receiving TAVR, valve type did not predict stroke at 1 year. The predominant drivers of stroke were clinical variables: age, STS risk, and a history of stroke.

Since transcatheter aortic valve replacement (TAVR) is now being increasingly performed in patients at low surgical risk, mitigating procedural complications has become very relevant.^1^, ^2^, ^3^, ^4^ Stroke remains one of the most consequential adverse events after TAVR, given its association with disability, mortality, and long-term neurocognitive decline.^5^ Contemporary randomized trials and large registries report periprocedural stroke rates of 0.6% to 6.7%, with most modern cohorts showing rates of 2 to 3%.^5^ This large variance in stroke risk reflects selection bias and other confounders. Despite advances in the field, stroke rates have plateaued in the last few years.

There are multiple mechanisms of stroke after TAVR. These range from embolization of valvular or aortic debris during the procedure to new-onset atrial fibrillation. Even subclinical leaflet thrombosis or hypoattentuated leaflet thickening has been implicated in stroke after TAVR.^5^^,^^6^ Although several patient-level factors and anatomical features have been associated with stroke risk, findings across studies remain inconsistent, often limited by small sample size or selection bias and other confounding variables.^7^^,^^8^

The influence of valve type—balloon-expandable valves (BEV) vs self-expanding valves (SEV)—on stroke risk remains unresolved. Some meta-analyses have suggested a higher stroke risk with BEVs,^9^^,^^10^ while others—including large registries and randomized trials—show no difference or even a lower stroke risk with BEVs.^4^^,^^11^^,^^12^ Additionally, previous studies have primarily examined stroke within 30 days of TAVR and do not fully capture current practice.^13^

To address this knowledge gap, an analysis of a large, contemporary multicenter cohort of TAVR patients within a nonprofit health system, CommonSpirit Health, between 2021 and 2023 was done using data from the Society of Thoracic Surgeons (STS)/American College of Cardiology (ACC) Transcatheter Valve Therapy (TVT) Registry.

## Methods

### Data source

All data for this analysis were obtained from the STS/ACC TVT Registry. The TVT Registry captures information on patient demographics, comorbidities, echocardiographic indices, procedural characteristics, and in-hospital, 30-day, and 1-year outcomes for consecutive patients undergoing commercial TAVR at participating sites across the United States.^14^ The Centers for Medicare & Medicaid Services (CMS) national coverage determination for TAVR mandates participation in a national registry for all Food and Drug Administration–approved devices, with the TVT Registry serving as the sole registry fulfilling this requirement.^15^ Data completeness, consistency, and quality are ensured through ongoing monitoring by the Duke Clinical Research Institute and the National Cardiovascular Data Registry, as well as an annual third-party random audit of approximately 10% of submitted records.^16^ The registry is approved by the central Institutional Review Board (IRB) with a waiver of informed consent under the Common Rule.

For this study, we included all patients in the TVT Registry who underwent TAVR within the CommonSpirit Health system between January 2021 and February 2023. The CommonSpirit Health system includes 146 hospitals that perform approximately 3,500 TAVRs annually. The creation and use of the CommonSpirit TAVR research database were approved and overseen by the CommonSpirit Health Cardiovascular Service Line and the CommonSpirit Research Institute IRB, which serves as the IRB of record for this project.

### Outcomes and definitions

The primary outcome of this study was the incidence of stroke following TAVR. Secondary outcomes included major bleeding, myocardial infarction, new-onset atrial fibrillation, cardiac surgery, hospital readmission (overall, valve-related, and non–valve-related), and all-cause mortality, as reported within the STS/ACC TVT Registry. Standardized definitions for all adverse events and clinical outcomes followed the TVT Registry Data Coder Dictionary, which incorporates Valve Academic Research Consortium–based criteria.^17^^,^^18^ Clinical outcomes in the TVT Registry are captured during the index hospitalization and at 30 days and 1 year after the procedure. To ensure complete ascertainment of postdischarge events, all outcomes were supplemented through linkage with the CMS claims database, which provides longitudinal follow-up for Medicare beneficiaries.

The primary exposure of interest was the type of transcatheter heart valve implanted, categorized as BEV or SEV. Valve classification in the STS/ACC TVT Registry is based on device-specific identifiers recorded at the time of implantation. We hypothesized that SEV would be associated with higher stroke rates compared with BEV following TAVR.

### Statistical analysis

Continuous variables were reported as mean ± SD and compared using the independent sample *t*-test, Mann-Whitney *U* test, or analysis of variance, as appropriate. Categorical variables were expressed as absolute counts and percentages and compared using the chi-square or Fisher exact test, as appropriate.

Time-to-event methods were used for the primary outcome. Stroke-free survival was summarized using Kaplan-Meier estimates and compared between valve groups using the log-rank test.

To adjust for baseline differences between valve types, inverse probability of treatment weighting (IPTW) was performed using stabilized weights derived from a propensity score estimated via logistic regression incorporating prespecified demographic, clinical, echocardiographic, and procedural covariates (listed in Supplemental Table 1).^19^ Balance between groups was assessed using standardized mean differences, with an absolute standardized mean differences <0.10 indicating acceptable covariate balance.^20^ Weight diagnostics (percentiles and effective sample size) were examined to evaluate positivity and weight stability (Supplemental Table 2). Time-to-event analyses were performed to evaluate 1-year stroke incidence. The primary adjusted analysis used Cox proportional hazards models with IPTW to calculate HRs and 95% CIs, with robust (sandwich) variance estimators used for inference. Proportional hazards assumptions were assessed and were not violated.

To account for potential clustering at the hospital level, sensitivity analyses were performed using robust variance estimators with hospital specified as the clustering variable.

Because death may preclude the occurrence of stroke, a competing-risk sensitivity analysis was performed using IPTW-weighted Fine–Gray subdistribution hazard models, treating death without prior stroke as the competing event. Subdistribution HRs and 95% CIs were reported.

A *P* value ≤0.05 was considered statistically significant. Analyses were conducted using MedCalc Statistical Software version 12.7.7 (MedCalc Software).

## Results

### Study cohort

The study cohort consisted of 6,663 patients who underwent TAVR at CommonSpirit Health hospital sites between January 2021 and February 2023 and were recorded in the STS/ACC TVT Registry. Of these, 5,445 patients (81.7%) received a BEV, and 1,218 patients (18.3%) received a SEV.

### Clinical characteristics

As shown in Table 1, patients treated with SEV were significantly older than those receiving BEV (79.2 ± 7.7 vs 73.2 ± 11.0 years; *P* < 0.001). A higher proportion of SEV patients were female (56.7% vs 37.5%; *P* < 0.001) and had a greater STS risk score (4.5 ± 3.8 vs 4.0 ± 3.5; *P* < 0.001). Peripheral arterial disease was less frequent in the SEV cohort (16.3% vs 23.2%; *P* < 0.001), whereas congestive heart failure was more prevalent (70.1% vs 65.7%; *P* = 0.004). Patients undergoing SEV implantation were also less likely to present with NYHA functional class III or IV symptoms compared with those receiving BEV (59.8% vs 66.2%; *P* < 0.001).

**Table 1:** Baseline and Clinical Characteristics

	Total (N = 6,663)	BEV (n = 5,445)	SEV (n = 1,218)	*P* Value
Age, y	74.3 ± 10.7	73.2 ± 11.0	79.2 ± 7.7	<0.001
Body mass index, kg/m^2^	29.7 ± 9.9	29.7 ± 9.9	29.6 ± 10.0	0.752
Female	2,735 (41.0)	2044 (37.5)	691 (56.7)	<0.001
STS risk score	4.1 ± 3.6	4.0 ± 3.5	4.5 ± 3.8	<0.001
KCCQ12 score	48.5 ± 26.2	48.3 ± 26.3	49.6 ± 25.6	0.111
Race				
Caucasian	6,077 (91.2)	4,967 (91.2)	1,110 (91.1)	0.866
African American	174 (2.6)	135 (2.5)	39 (3.2)	0.183
Asian	118 (1.8)	96 (1.8)	22 (1.8)	1.00
American Indian	26 (0.4)	24 (0.4)	2 (0.2)	0.252
Other/unknown	268 (4.0)	223 (4.1)	45 (3.7)	0.573
Hispanic ethnicity	491 (7.4)	411 (7.5)	80 (6.6)	0.262
Comorbidities				
Hypertension	5,979 (89.7)	4,864 (89.3)	1,115 (91.5)	0.025
Diabetes mellitus	2,468 (37.0)	2029 (37.3)	439 (36.0)	0.444
Prior stroke	1,241 (18.6)	1,007 (18.5)	234 (19.2)	0.589
Atrial fibrillation	2,263 (34.0)	1878 (34.5)	385 (31.6)	0.059
Prior myocardial infarction	1,045 (15.7)	871 (16.0)	174 (14.3)	0.150
Prior PCI	1847 (27.7)	1,534 (28.2)	313 (25.7)	0.087
Prior CABG	884 (13.3)	716 (13.1)	168 (13.8)	0.581
Carotid artery stenosis	1,015 (15.2)	846 (15.5)	169 (13.9)	0.157
Peripheral arterial disease	1,461 (21.9)	1,263 (23.2)	198 (16.3)	<0.001
Congestive heart failure	4,433 (66.5)	3,579 (65.7)	854 (70.1)	0.004
NYHA functional class III or IV	4,335 (65.1)	3,607 (66.2)	728 (59.8)	<0.001
Etiology of aortic valve disease				
Degenerative	6,313 (94.7)	5,213 (95.7)	1,100 (90.3)	<0.001
Endocarditis	8 (0.1)	7 (0.1)	1 (0.08)	1.00
Rheumatic	14 (0.2)	12 (0.2)	2 (0.16)	0.967
Other	328 (4.9)	213 (3.9)	115 (9.4)	<0.001

Values are mean ± SD or n (%).

BEV = balloon-expandable valve; CABG = coronary artery bypass graft; KCCQ = Kansas City Cardiomyopathy Questionnaire; PCI = percutaneous coronary intervention; SEV = self-expanding valve; STS = Society of Thoracic Surgeons.

The proportion of bicuspid aortic valves was (7.5% vs 7.0%; *P* = 0.591) (Table 2). Annular calcium was frequently present but did not differ significantly between groups (72.9% in BEV vs 75.0% in SEV; *P* = 0.154).

**Table 2:** Baseline Echocardiography

	Total (N = 6,663)	BEV (n = 5,445)	SEV (n = 1,218)	*P* Value
LVEF, %	56.6 ± 11.4	56.3 ± 11.6	58.0 ± 10.4	<0.001
Tricuspid aortic valve	5,942 (89.2)	4,864 (89.3)	1,078 (88.5)	0.432
Bicuspid aortic valve	492 (7.4)	407 (7.5)	85 (7.0)	0.591
Other/unknown	229 (3.4)	174 (3.2)	55 (4.5)	0.028
Annular calcium	4,883 (73.3)	3,970 (72.9)	913 (75.0)	0.154
Aortic regurgitation				
None/trace/trivial	3,250 (48.8)	2,653 (48.7)	597 (49.0)	0.879
Mild	2,273 (34.1)	1862 (34.2)	411 (33.7)	0.789
Moderate	927 (13.9)	772 (14.2)	155 (12.7)	0.201
Severe	213 (3.2)	158 (2.9)	55 (4.5)	0.005
Aortic valve area	0.76 ± 0.26	0.76 ± 0.26	0.76 ± 0.27	1.00
Aortic valve mean gradient, mm Hg	42.3 ± 13.8	42.4 ± 13.8	41.8 ± 14.0	0.175
Aortic valve peak gradient, mm Hg	69.1 ± 21.7	69.1 ± 21.6	68.9 ± 22.0	0.774
Aortic valve peak velocity, m/s	4.1 ± 0.7	4.1 ± 0.7	4.1 ± 0.8	1.00
Mitral valve regurgitation				
None/trace/trivial	3,440 (51.6)	2,934 (53.9)	506 (41.5)	<0.001
Mild	2,236 (33.6)	1728 (31.7)	508 (41.7)	<0.001
Moderate	786 (11.8)	630 (11.6)	156 (12.8)	0.245
Moderate-severe	98 (1.5)	76 (1.4)	22 (1.8)	0.345
Severe	103 (1.5)	77 (1.4)	26 (2.1)	0.087
Mitral valve area, cm^2^	2.8 ± 1.2	2.8 ± 1.2	2.7 ± 1.2	0.009
Mitral valve mean gradient, mm Hg	4.4 ± 8.3	4.4 ± 8.6	4.4 ± 6.7	1.00
Primary mitral regurgitation	1,079 (16.2)	864 (15.9)	215 (17.7)	0.138
Secondary mitral regurgitation	170 (2.6)	143 (2.6)	27 (2.2)	0.472
Tricuspid valve regurgitation				
None/trace/trivial	3,218 (48.3)	2,710 (49.8)	508 (41.7)	<0.001
Mild	2,413 (36.2)	1936 (35.6)	477 (39.2)	0.020
Moderate	873 (13.1)	667 (12.2)	206 (16.9)	<0.001
Severe	159 (2.4)	132 (2.4)	27 (2.2)	0.745

Values are mean ± SD or n (%).

LVEF = left ventricular ejection fraction; other abbreviations as in Table 1.

### Procedural details

As detailed in Table 3, use of general anesthesia was more common in SEV procedures compared with BEV (33.0% vs 24.1%; *P* < 0.001), although overall procedure length was similar between groups (71.4 ± 46.4 vs 69.7 ± 45.7 minutes; *P* = 0.247). Fluoroscopy time was significantly longer among SEV cases (17.5 ± 11.5 vs 14.0 ± 7.7 minutes; *P* < 0.001). Embolic protection devices were used infrequently but more often in SEV procedures (5.5% vs 4.0%; *P* = 0.024). SEV patients were also less likely to undergo urgent procedures (6.4% vs 12.3% for BEV; *P* < 0.001). Femoral access was the predominant access route in both groups with no significant difference (96.7% SEV vs 96.4% BEV; *P* = 0.608). Postimplantation hemodynamics showed a higher mean gradient in BEV recipients compared with SEV (5.0 ± 4.2 vs 5.8 ± 4.0 mm Hg; *P* < 0.001).

**Table 3:** Procedural Details

	Total (N = 6,663)	BEV (n = 5,445)	SEV (n = 1,218)	*P* Value
Anesthesia type				
Moderate	4,677 (70.2)	3,892 (71.5)	785 (64.4)	<0.001
General	1712 (25.7)	1,310 (24.1)	402 (33.0)	<0.001
Deep	270 (4.1)	240 (4.4)	30 (2.5)	0.002
Valve type				
Sapien 3	1,153 (17.3)	1,153 (21.2)	-	NA
Sapien 3 Ultra	4,268 (64.1)	4,268 (78.4)	-	NA
Sapien 3 Ultra Resilia	12 (0.2)	12 (0.2)	-	NA
Sapien XT	12 (0.2)	12 (0.2)	-	NA
CoreValve Evolut R	32 (0.5)	-	32 (2.6)	NA
Evolut FX	382 (5.7)	-	382 (31.4)	NA
Evolut PRO	29 (0.4)	-	29 (2.4)	NA
Evolut Pro Plus	775 (11.6)	-	775 (63.6)	NA
Device diameter, mm				
20	142 (2.1)	142 (2.6)	-	NA
23	1825 (27.4)	1733 (31.8)	92 (7.6)	NA
26	2,981 (44.7)	2,469 (45.3)	512 (42.0)	NA
29	1,530 (23.0)	1,099 (20.2)	431 (35.4)	NA
34	183 (2.7)	-	183 (15.0)	NA
Procedure length, min	70.0 ± 45.8	69.7 ± 45.7	71.4 ± 46.4	0.247
Fluoroscopy time, min	14.6 ± 8.6	14.0 ± 7.7	17.5 ± 11.5	<0.001
Contrast volume	80.3 ± 54.3	78.6 ± 54.3	88.1 ± 53.9	<0.001
Embolic protection	285 (4.3)	218 (4.0)	67 (5.5)	0.024
Procedure status				
Elective	5,898 (88.5)	4,758 (87.4)	1,140 (93.6)	<0.001
Urgent	750 (11.3)	672 (12.3)	78 (6.4)	<0.001
Emergent/salvage	15 (0.2)	15 (0.3)	0 (0)	0.134
Access method				
Femoral	6,425 (96.4)	5,247 (96.4)	1,178 (96.7)	0.608
Subclavian	45 (0.7)	38 (0.7)	7 (0.6)	0.779
Transeptal	2 (0.03)	2 (0.04)	0 (0)	>0.999
Transcaval	3 (0.05)	3 (0.06)	0 (0)	0.942
Transapical	5 (0.08)	5 (0.09)	0 (0)	0.632
Transaxillary	36 (0.5)	28 (0.5)	8 (0.7)	0.691
Transcarotid	135 (2.0)	114 (2.1)	21 (1.7)	0.475
Other	12 (0.2)	8 (0.1)	4 (0.3)	0.329
Concomitant PCI	49 (0.7)	45 (0.8)	4 (0.3)	0.065
Postimplantation mean gradient	5.1 ± 4.2	5.0 ± 4.2	5.8 ± 4.0	<0.001

Values are n (%) or mean ± SD.

NA = not applicable; other abbreviations as in Table 1.

### Thirty-day echocardiographic data

The distribution of postprocedural aortic regurgitation differed between groups: patients treated with BEV were more likely to have none, trace, or trivial regurgitation (86.3% vs 75.0%; *P* < 0.001), whereas SEV patients demonstrated higher rates of mild (23.6% vs 13.0%; *P* < 0.001) and moderate regurgitation (1.3% vs 0.7%; *P* = 0.031) (Table 4). Hemodynamic performance also varied by valve type, with SEV associated with a larger aortic valve area at 30 days (2.0 ± 0.6 cm^2^ vs 1.7 ± 0.6 cm^2^; *P* < 0.001) and a lower mean gradient (8.2 ± 5.0 mm Hg vs 12.6 ± 5.8 mm Hg; *P* < 0.001).

**Table 4:** Thirty-Day Echocardiography

	Total (N = 6,663)	BEV (n = 5,445)	SEV (n = 1,218)	*P* Value
LVEF, %	57.8 ± 9.9	57.6 ± 10.0	58.7 ± 9.1	<0.001
Aortic regurgitation				
None/trace/trivial	5,615 (84.3)	4,701 (86.3)	914 (75.0)	<0.001
Mild	995 (14.9)	708 (13.0)	287 (23.6)	<0.001
Moderate	52 (0.8)	36 (0.7)	16 (1.3)	0.031
Severe	1 (0.02)	0 (0)	1 (0.08)	0.412
Aortic valve area	1.8 ± 0.6	1.7 ± 0.6	2.0 ± 0.6	<0.001
Aortic valve mean gradient, mm Hg	11.8 ± 5.9	12.6 ± 5.8	8.2 ± 5.0	<0.001
Paravalvular leak				
Mild	999 (15.0)	717 (13.2)	282 (23.2)	<0.001
Moderate	42 (0.6)	32 (0.6)	10 (0.8)	0.465
Severe	0 (0)	0 (0)	0 (0)	-

Values are mean ± SD or n (%).

Abbreviations as in Tables 1 and 2.

### Clinical outcomes

A total of 87 patients experienced stroke within the study period, corresponding to an incidence of 1.3% across the study population (Table 5). Stroke occurred in 1.2% of BEV recipients (64 of 5,445) and 1.9% of SEV recipients (23 of 1,218), a difference that was not statistically significant (*P* = 0.051). Major bleeding was observed in 93 patients (1.4%), readmission in 1,198 patients (18.0%), new-onset atrial fibrillation in 139 patients (2.1%), and death in 432 patients (6.5%). For each of these secondary outcomes, rates were similar between BEV and SEV groups, with no statistically significant differences observed.

**Table 5:** Clinical Outcomes

	Total (N = 6,663)	BEV (n = 5,445)	SEV (n = 1,218)	*P* Value
Stroke	87 (1.3)	64 (1.2)	23 (1.9)	0.051
Major bleeding	93 (1.4)	69 (1.3)	24 (2.0)	0.079
Readmission	1,198 (18.0)	993 (18.2)	205 (16.8)	0.265
Valve-related	52 (0.8)	42 (0.8)	10 (0.8)	1.00
Non valve-related	1,146 (17.2)	951 (17.5)	195 (16.0)	0.240
Myocardial infarction	37 (0.6)	32 (0.6)	5 (0.4)	0.590
Cardiac surgery	41 (0.6)	37 (0.7)	4 (0.3)	0.221
Atrial fibrillation (new onset)	139 (2.1)	114 (2.1)	25 (2.1)	1.00
Death	432 (6.5)	348 (6.4)	84 (6.9)	0.560

Values are n (%).

Abbreviations as in Table 1.

**Figure 1:**
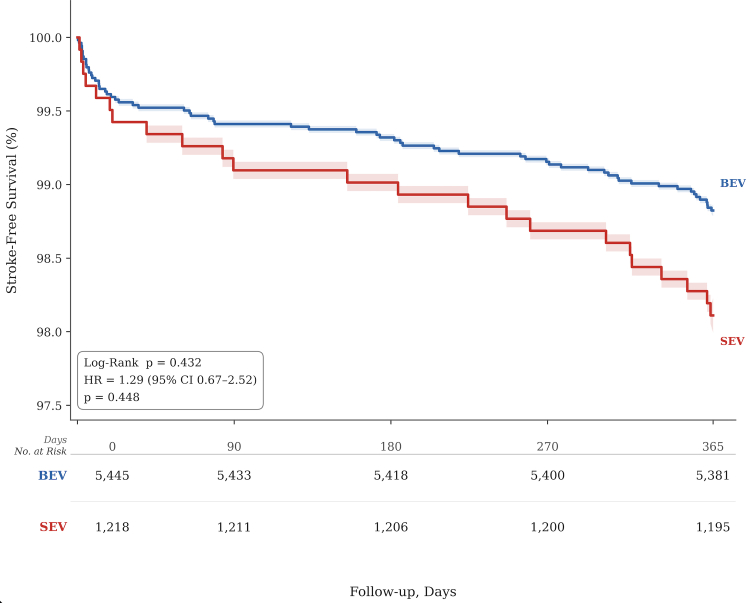
**Stroke-Free Survival After Transcatheter Aortic Valve Replacement by Valve Type** Kaplan-Meier curves comparing stroke-free survival between balloon-expandable valves and self-expanding valves over 1 year. BEV = balloon-expandable valve; SEV = self-expanding valve.

As shown in Figure 1, Kaplan-Meier curves for stroke-free survival remained comparable over the follow-up period, with no significant separation observed (log-rank *P* = 0.448).

As shown in Supplemental Table 1 and Supplemental Figure 1, IPTW adjustment resulted in effective adjustment between groups. In addition, weight diagnostics were examined to assess positivity and model stability. The distribution of stabilized weights demonstrated a median of 0.96 (1st–99th percentile: 0.41-2.08), with a maximum weight of 4.87. The effective sample size after weighting was 6,189, indicating minimal loss of precision and no evidence of extreme weights (Supplemental Table 2).

Table 6 presents the IPTW analysis evaluating independent predictors of stroke. Compared to BEV, SEV was not associated with a significant difference in stroke risk (adjusted HR [aHR]: 1.54; 95% CI: 0.79-2.68; *P* = 0.294). Several variables demonstrated significant associations with stroke, including increasing age (aHR: 1.11 per year; 95% CI: 1.05-1.16; *P* < 0.001), prior stroke (aHR: 2.08; 95% CI: 1.26-3.34; *P* = 0.003), higher STS risk score (aHR: 2.94; 95% CI: 1.63-4.48; *P* < 0.001), lower body mass index (BMI) (aHR: 0.93 per kg/m^2^; 95% CI: 0.89-0.97; *P* = 0.001), and femoral artery access (aHR: 0.39; 95% CI: 0.16-0.98; *P* = 0.042). Atrial fibrillation (aHR: 1.44; 95% CI: 0.93-2.23; *P* = 0.102) and the use of embolic protection devices (aHR: 0.47; 95% CI: 0.11-1.89; *P* = 0.309) were not statistically significant predictors of stroke.

**Table 6:** Independent Predictors of Stroke (IPTW-Adjusted Analysis)

	HR (95% CI)	*P* Value
Self-expanding valve	1.54 (0.79-2.68)	0.294
Balloon-expandable valve	0.65 (0.37-1.27)	0.294
Age	1.11 (1.05-1.16)	<0.001
Prior stroke	2.08 (1.26-3.34)	0.003
Aortic peak velocity, baseline	0.69 (0.50-1.00)	0.036
Body mass index	0.93 (0.89-0.97)	0.001
Femoral artery access	0.39 (0.16-0.98)	0.042
Female sex	1.52 (0.97-2.42)	0.073
STS risk score	2.94 (1.63-4.48)	<0.001
Atrial fibrillation	1.44 (0.93-2.23)	0.102
Myocardial infarction	1.18 (0.69-2.02)	0.552
Embolic protection	0.47 (0.11-1.89)	0.309
Bicuspid aortic valve	1.57 (0.72-3.41)	0.266
Prior PCI	1.07 (0.68-1.70)	0.741
Hypertension	1.48 (0.69-3.09)	0.312
Elective procedure	1.34 (0.66-2.89)	0.461
LVEF (%)	2.12 (0.32-15.02)	0.581
≥ moderate aortic insufficiency	1.27 (0.75-2.18)	0.398
Carotid artery stenosis	1.09 (0.61-1.92)	0.634
KCCQ-12 baseline score	0.99 (0.97-1.01)	0.240
Nondegenerative etiology	1.29 (0.46-3.54)	0.628
Congestive heart failure	0.98 (0.62-1.55)	0.934
Moderate sedation	0.88 (0.54-1.38)	0.595
Annular calcium	1.04 (0.64-1.67)	0.879
Prior CABG	0.94 (0.52-1.74)	0.840
NYHA functional class III or IV symptoms	1.02 (0.65-1.63)	0.930
Diabetes mellitus	0.92 (0.58-1.46)	0.726
Peripheral arterial disease	1.08 (0.67-1.82)	0.766

IPTW = inverse probability of treatment weighting; other abbreviations as in Tables 1 and 2.

In a sensitivity analysis restricted to clinically prespecified predictors (age, prior stroke, STS risk score, and access route), the association between valve type and stroke remained unchanged (aHR: 1.39; 95% CI: 0.78-3.01; *P* = 0.536).

In a sensitivity analysis accounting for clustering at the hospital level using robust variance estimators, the association between valve type and stroke remained unchanged (aHR: 1.57; 95% CI: 0.96-2.58; *P* = 0.074). Moreover, in competing-risk analysis accounting for death as a competing event, the 1-year cumulative incidence of stroke was 1.10% (95% CI: 0.85-1.41) in the BEV group and 1.72% (95% CI: 1.07-2.55) in the SEV group (absolute difference 0.62%; *P* = 0.063). IPTW-adjusted competing-risk analysis showed that valve type was not significantly associated with 1-year stroke (subdistribution HR: 1.65; 95% CI: 0.92-2.64; *P* = 0.122). Given the low number of stroke events, associations for individual covariates should be interpreted as exploratory rather than definitive causal effects.

## Discussion

The principal findings of this contemporary multicenter, retrospective analysis of a large integrated health system data set within the STS-TVT registry evaluating stroke after TAVR are as follows:

1. In the real world, balloon expandable valves are the predominant platform of choice. In this registry, they were used in 81.7% of the cases, with SEV used in the remaining 18.3%.
2. After adjustment and propensity matching, valve type was not an independent predictor of stroke after TAVR. Although the group receiving the SEV had more strokes numerically, this difference of 0.7% did not achieve statistical significance.
3. The strongest predictors of stroke after TAVR were STS risk score, prior stroke, age, and use of alternative access.
4. Atrial fibrillation, use of cerebral embolic protection (CEP) devices, and low left ventricular ejection fraction were not associated with stroke risk in this cohort (Central Illustration).

**Central Illustration:**
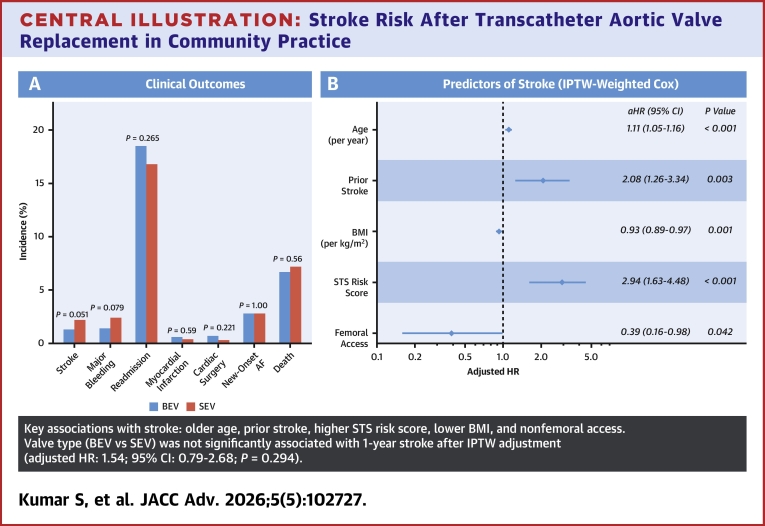
**Stroke Risk After Transcatheter Aortic Valve Replacement in Community Practice** In a contemporary, multicenter community cohort from the Society of Thoracic Surgeons/American College of Cardiology Transcatheter Valve Therapy Registry, 1-year stroke after transcatheter aortic valve replacement was infrequent (1.3%) and was not independently associated with valve type (balloon-expandable vs self-expanding). Stroke risk was driven primarily by patient-level clinical factors, including age, prior stroke, higher Society of Thoracic Surgeons risk score, and use of alternative access. BMI = body mass index; IPTW = inverse probability of treatment weighting; STS = Society of Thoracic Surgeons; TAVR = transcatheter aortic valve replacement; other abbreviations as in Figure 1.

It had been predicted that TAVR would not be a feasible mainstream option for patients with severe aortic stenosis because of the high risk of stroke. This has not been the case, with stroke incidence plateauing at 2 to 3% in contemporary studies.^3^^,^^21^ The stroke rate of 1.3% in this study is consistent with stroke rates observed in contemporary randomized trial settings. This lower-than-expected number might be related to limitations inherent to registry-based data collection, including self-reported bias and the lack of neurologist adjudication, both of which have been known to decrease the fidelity of stroke ascertainment.

Prior studies have identified severely reduced left ventricular ejection fraction (<30%) and longer procedure time as independent predictors of stroke.^22^ In this cohort, older age, higher STS risk score, prior stroke, and lower BMI were independent predictors of stroke.

The STS risk score, despite its drawbacks, is a powerful, validated tool for assessing overall risk in surgical aortic valve replacement and has been used routinely by heart teams to discuss mortality risk after TAVR. In contrast to 2 prior studies, in which the STS score was not an independent predictor of stroke after TAVR,^23^^,^^24^ this study found that the STS score was strongly predictive of stroke (aHR: 2.94). This study had a larger sample size compared to the 2 previously referenced studies. This enhanced power might be the reason for this difference, given the low frequency of strokes after TAVR.

The history of a prior stroke as a significant predictor of stroke after a TAVR is consistent with findings from prior studies^25^^,^^26^ and is biologically plausible.^27^^,^^28^ The association between increasing age and stroke risk observed aligns with prior reports, reflecting the reduced cerebrovascular resilience that accompanies advancing age.^29^

In interpreting the association between lower BMI and increased stroke risk, caution is warranted. Because this study is restricted to patients who underwent TAVR, the cohort represents a highly selected population shaped by referral patterns, procedural candidacy, frailty, and survival to the time of intervention. Conditioning on such selection factors may introduce collider bias, potentially inducing spurious associations between predictors and outcomes.^30^ Collider bias occurs when an analysis conditions on a variable that is a common effect of both the exposure and the outcome, inadvertently inducing a spurious association between them.^30^ Importantly, this phenomenon is not unique to BMI and may influence observed relationships for other variables in this model, including age and comorbidity burden. At the same time, excess adiposity is well established in the general population as a risk factor for ischemic stroke through mechanisms including hypertension, diabetes, inflammation, and prothrombotic states.^31^ Therefore, the inverse association observed in this TAVR cohort should not be interpreted as evidence that higher BMI is protective. Rather, it may reflect the complex interplay of selection effects, frailty, comorbidity patterns, and residual confounding inherent to procedural registries. Accordingly, BMI in this context should be interpreted as a hypothesis-generating prognostic marker within a selected TAVR population rather than as a causal determinant of stroke risk.

CEP was used in only 4.3% of cases in our cohort, limiting the ability to detect any meaningful clinical effect on stroke reduction. Consistent with prior randomized trials, CEP use was not associated with a lower stroke incidence in our analysis.^11^^,^^32^ This finding is not unexpected, as current devices such as Sentinel do not provide complete anatomic coverage—leaving territories such as the left vertebral system unprotected—and the overall stroke rate in contemporary TAVR practice is already low, making it challenging to demonstrate incremental benefit without a substantially larger sample size or an enriched population at higher baseline risk.

The majority of cases were performed using femoral access. It is known that femoral access is best for mitigating stroke risk.^33^ Like in previous studies, the use of alternative access was associated with a higher stroke risk. The overall number of cases in which alternative access was used was small, making comparisons in the types of alternative access and stroke risk unfeasible.

Despite clinical stroke rates being low after TAVR, the occurrence of a stroke is a random and unpredictable event. Analysis of large databases such as this allows for closing the knowledge gap on risks for stroke and for targeted intervention.

### Study limitations

This study has several limitations. First, as an observational analysis based on the STS/ACC TVT Registry, it is subject to residual confounding despite IPTW. Stroke ascertainment relied on site-reported data without systematic neurologist adjudication or routine neuroimaging, potentially leading to underreporting. Similarly, although missing data for baseline covariates were minimal (with <5% missingness for individual variables), the potential for residual bias related to missing data cannot be fully excluded. Second, although the multicenter, community-based design reflects real-world practice, findings from a single integrated health system may not be fully generalizable to all community or academic TAVR programs. Third, the low overall stroke incidence limited statistical power to detect modest associations, particularly for less frequently used strategies such as CEP. Fourth, detailed anatomic features (eg, aortic arch atheroma, calcium distribution) and routine postprocedural assessments for mechanisms such as subclinical leaflet thrombosis or new-onset atrial fibrillation were unavailable, precluding deeper mechanistic evaluation. Finally, while linkage with CMS claims enhances follow-up for Medicare beneficiaries, longer-term neurologic outcomes beyond 1 year could not be assessed. Similarly, although discharge medication data were available, including antiplatelet therapy at hospital discharge, detailed longitudinal data regarding medication refills, adherence, duration of therapy, and oral anticoagulation use were not available. At discharge, dual antiplatelet therapy with aspirin and a P2Y12 inhibitor was prescribed in 5,367 of 5,445 BEV patients (98.6%) and 1,195 of 1,218 SEV patients (98.1%) (*P* = 0.234), indicating no significant difference between groups at baseline. However, because longitudinal antithrombotic management and compliance data were unavailable, the potential impact of postdischarge medication patterns on stroke risk could not be fully assessed.

## Conclusions

In real-world settings, the incidence of clinical stroke after TAVR remains low, as reported in the randomized controlled trials. The choice of a transcatheter heart valve does not influence the risk of stroke after TAVR. The primary driver of stroke associated with TAVR remains patient-level clinical factors such as a history of prior stroke and Society of Thoracic Surgeons Predicted Risk of Mortality (STS PROM).

Perspectives **COMPETENCY IN MEDICAL KNOWLEDGE:** In a large, contemporary community-based cohort from the STS/ACC TVT Registry, the incidence of 1-year clinical stroke after TAVR was low (1.3%), and transcatheter valve platform (balloon-expandable vs self-expanding) was not independently associated with stroke risk after adjustment. Instead, stroke risk was driven predominantly by patient-level clinical factors, including increasing age, higher STS risk score, prior stroke, and use of alternative access.

**TRANSLATIONAL OUTLOOK:** Although stroke after TAVR is now infrequent in contemporary practice, it remains a devastating and unpredictable complication. These findings suggest that risk stratification and prevention strategies should focus less on valve selection and more on identifying high-risk clinical phenotypes. Future studies should incorporate standardized neurologic adjudication and granular mechanistic phenotyping, develop validated risk prediction tools for community practice, and evaluate targeted preventive strategies (eg, tailored antithrombotic regimens, rhythm surveillance, and selective embolic protection) in patients at highest baseline risk to further reduce stroke after TAVR.

## Funding support and author disclosures

The authors have reported that they have no relationships relevant to the contents of this paper to disclose.
