# Supplementary material for: Stroke Risk After TAVR: Balloon-Expandable vs Self-Expanding Valves in Community Practice
Source: JACC Adv. 2026 Apr 9;5(5):102727. doi: 10.1016/j.jacadv.2026.102727 (PMC13091329; doi:10.1016/j.jacadv.2026.102727)
Supplement: Supplemental Material [file mmc1.docx]

**Supplemental Appendix**

**Supplemental Table 1. Baseline Characteristics, Baseline Echocardiography, and Procedural Details in the IPTW-Adjusted Cohort***

|  | **BEV**  **(N = 5445)** | **SEV**  **(N = 1218)** | **\|SMD\|** |
| --- | --- | --- | --- |
| **Age** | 78.7 ± 8.2 | 78.9 ± 8.3 | 0.018 |
| **Body Mass Index** | 29.5 ± 8.2 | 30.8 ± 17.8 | 0.092 |
| **Female** | 41.3% | 40.1% | 0.025 |
| **STS Risk Score** | 4.0 ± 3.5 | 4.5 ± 3.8 | 0.008 |
| **KCCQ12 Baseline Score** | 48.6 ± 26.4 | 49.2 ± 25.2 | 0.026 |
| **Hypertension** | 89.9% | 88.5% | 0.047 |
| **Diabetes Mellitus** | 36.6% | 38.5% | 0.039 |
| **Atrial Fibrillation** | 32.8% | 33.6% | 0.018 |
| **Chronic Lung Disease** | 29.1% | 28.0% | 0.023 |
| **Prior MI** | 15.7% | 15.4% | 0.008 |
| **Prior Stroke** | 18.5% | 19.2% | 0.013 |
| **Prior CABG** | 13.4% | 13.2% | 0.006 |
| **Congestive Heart Failure** | 66.4% | 67.9% | 0.031 |
| **NYHA Class III or IV** | 64.9% | 65.3% | 0.007 |
| **Peripheral Arterial Disease** | 22.2% | 20.6% | 0.04 |
| **Carotid Artery Stenosis** | 15.0% | 17.2% | 0.059 |
| **LVEF**, % | 57.1 ± 11.0 | 57.1 ± 12.0 | 0.001 |
| **≥ Moderate Aortic Regurgitation** | 16.8% | 18.2% | 0.038 |
| **Non-Tricuspid Aortic Valve** | 10.8% | 11.6% | 0.025 |
| **Annular Calcium** | 72.9% | 74.7% | 0.039 |
| **Aortic Valve Area** | 0.8 ± 0.3 | 0.8 ± 0.3 | 0.029 |
| **Aortic Valve Peak Velocity** | 4.1 ± 0.7 | 4.1 ± 0.7 | 0.003 |
| **Aortic Valve Peak Gradient** | 69.0 ± 21.6 | 69.1 ± 21.9 | 0.007 |
| **Non-Degenerative Etiology** | 5.6% | 4.4% | 0.044 |
| **Non-Elective Procedure** | 11.7% | 10.9% | 0.028 |
| **≥ Moderate Sedation** | 68.7% | 70.6% | 0.041 |
| **Femoral Artery Access** | 96.7% | 95.7% | 0.052 |
| **Concomitant PCI** | 0.7% | 1.1% | 0.043 |
| **Embolic Protection Device** | 4.4% | 3.6% | 0.041 |

*Variables incorporated in the IPTW-Adjusted model: age, sex, STS risk score, BMI, race/ethnicity, prior stroke, atrial fibrillation, diabetes, hypertension, peripheral arterial disease, carotid artery stenosis, prior PCI, prior CABG, congestive heart failure, NYHA class, LVEF, aortic valve morphology, annular calcium, aortic valve gradients and area, etiology of valve disease, procedure status (elective/urgent), access site, and use of embolic protection

| **Supplemental Table 2. Stabilized Inverse Probability of Treatment Weight Diagnostics and Effective Sample Size** |
| --- |

| **Statistic** | **Stabilized IPTW** |
| --- | --- |
| Original sample size | 6,663 |
| Effective sample size (ESS) | 6,189 |
| Minimum | 0.24 |
| 1st percentile | 0.41 |
| 5th percentile | 0.62 |
| Median | 0.96 |
| 95th percentile | 1.40 |
| 99th percentile | 2.08 |
| Maximum | 4.87 |

**Supplemental Figure 1. Standard Mean Differences Before and After Adjustment**

**
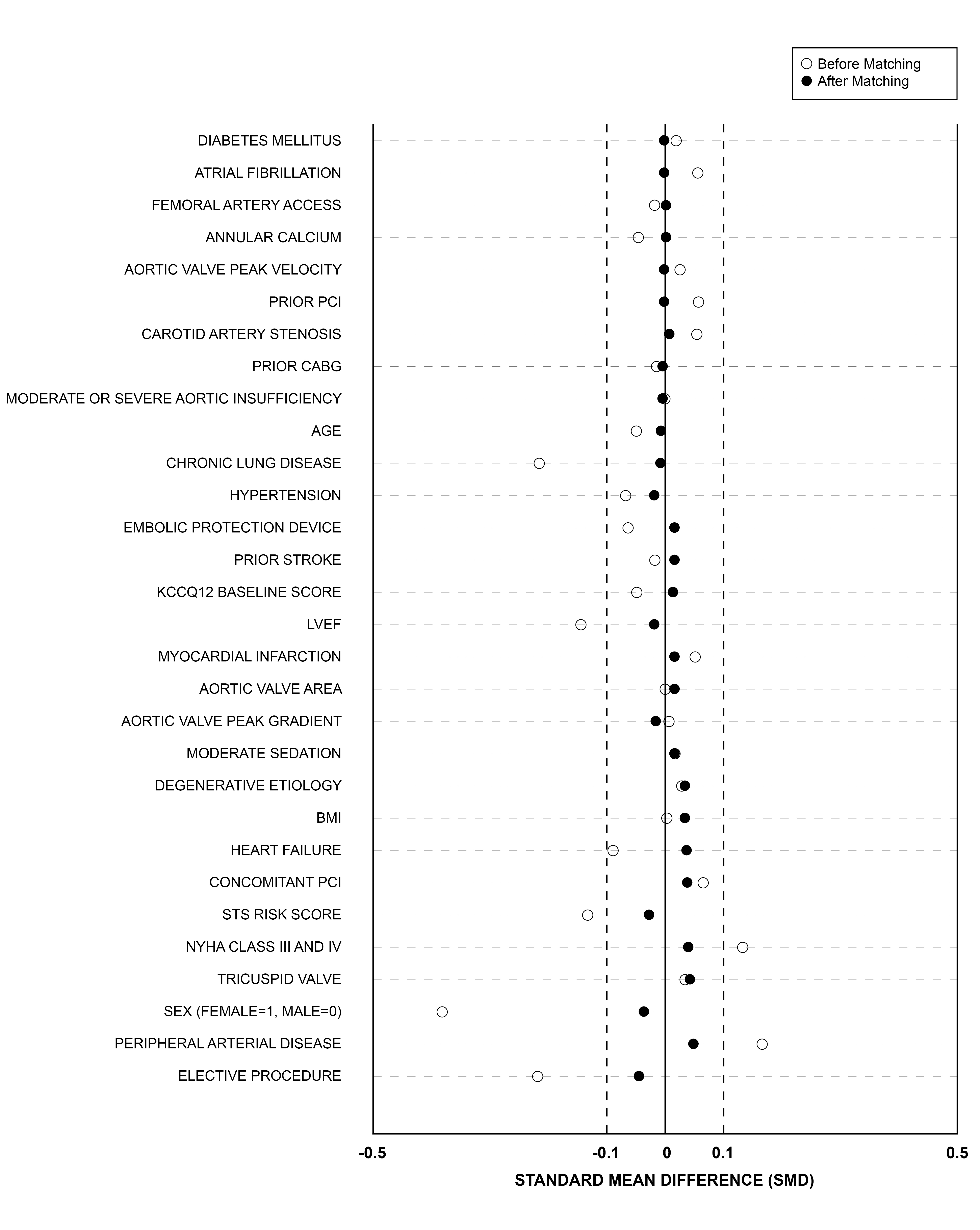
**
